# Supplementary material for: The prevalence of undernutrition and associated risk factors in people with tuberculosis in Lao People’s Democratic Republic
Source: PLoS One. 2025 Jun 20;20(6):e0324838. doi: 10.1371/journal.pone.0324838 (PMC12180643; doi:10.1371/journal.pone.0324838)
Supplement: S1 Table — (DOCX) [file pone.0324838.s001.docx]

| **Risk factor** | | **N** | **People with BMI < 18.5 n (%)** | **Crude odds ratio (95% CI)** | **Adjusted odds ratio (95% CI)** |
| --- | --- | --- | --- | --- | --- |
| Age group | 35-44 | 42 | 14 (33.3) | Reference | Reference |
|  | 0-14 | 1 | 0 (0.0) | N/A | N/A |
|  | 15-24 | 28 | 18 (66.7) | 4.00 (1.47-11.58) | 6.92 (2.25-23.20) |
|  | 25-34 | 56 | 28 (49.1) | 1.93 (0.85-4.49) | 2.26 (0.91-5.78) |
|  | 45-54 | 58 | 14 (24.1) | 0.64 (0.26-1.54) | 0.79 (0.30-2.06) |
|  | 55-64 | 62 | 22 (35.5) | 1.10 (0.48-2.55) | 0.95 (0.38-2.37) |
|  | 65+ | 65 | 31 (47.7) | 1.82 (0.82-4.15) | 1.79 (0.75-4.41) |
| Sex | Female | 117 | 47 (40.2) | Reference | - |
|  | Male | 195 | 80 (41.0) | 1.04 (0.65-1.66) | - |
| Marital status | Married | 189 | 69 (36.5) | Reference | - |
|  | Single | 76 | 41 (53.9) | 2.04 (1.19-3.51) | - |
|  | Divorced/separated/widowed | 47 | 17 (36.2) | 0.99 (0.50-1.90) | - |
| Smoker | No smoking experience | 157 | 61 (39.1) | Reference | - |
|  | Current smoker | 32 | 17 (53.1) | 1.77 (0.82-3.83) | - |
|  | Ex-smoker | 123 | 49 (39.5) | 1.02 (0.63-1.65) | - |
| Alcohol use | Daily | 25 | 14 (53.8) | Reference | - |
|  | Weekly | 31 | 12 (38.7) | 0.54 (0.18-1.55) | - |
|  | Monthly | 30 | 15 (50.0) | 0.86 (0.30-2.46) | - |
|  | Rarely/Never | 226 | 86 (38.2) | 0.53 (0.23-1.20) | - |
| Drug resistance status | TB (first-line treatment) | 296 | 117 (39.4) | Reference | Reference |
|  | MDR/RR-TB | 16 | 10 (66.7) | 3.08 (1.07-10.09) | 3.21 (1.00-11.81) |
| Household size | ≥5 | 158 | 68 (42.8) | Reference | - |
|  | <5 | 154 | 59 (38.6) | 0.84 (0.53-1.32) | - |
| Education level | No education | 32 | 16 (50.0) | Reference | - |
|  | Primary | 83 | 38 (45.8) | 0.84 (0.37-1.92) | - |
|  | Lower/higher secondary | 137 | 52 (37.7) | 0.60 (0.28-1.32) | - |
|  | Diploma or higher, vocational, other | 60 | 21 (35.6) | 0.55 (0.23-1.32) | - |
| Employment status before having TB | Unemployed | 79 | 37 (46.2) | Reference | - |
|  | Formal paid work | 48 | 14 (29.2) | 0.48 (0.22-1.01) | - |
|  | Informal paid work | 146 | 56 (38.4) | 0.72 (0.42-1.26) | - |
|  | Retired/student/housework/other | 39 | 20 (52.6) | 1.29 (0.60-2.82) | - |
| Main income earner | No | 191 | 82 (42.9) | Reference | - |
|  | Yes | 103 | 39 (37.9) | 0.81 (0.49-1.32) | - |
|  | Equal contributor | 18 | 6 (33.3) | 0.66 (0.22-1.79) | - |
| Household income quintile | Fifth (highest) | 63 | 24 (38.1) | Reference | - |
|  | Fourth | 85 | 35 (41.2) | 1.14 (0.58-2.23) | - |
|  | Third | 45 | 15 (32.6) | 0.79 (0.35-1.74) | - |
|  | Second | 64 | 27 (42.2) | 1.19 (0.58-2.42) | - |
|  | First (lowest) | 55 | 26 (48.1) | 1.51 (0.72-3.17) | - |
| Site and diagnosis of TB | Pulmonary, bacteriologically confirmed | 225 | 90 (40.2) | Reference | - |
|  | Pulmonary, bacteriologically unconfirmed (clinically diagnosed) | 70 | 31 (43.7) | 1.15 (0.67-1.98) | - |
|  | Extrapulmonary | 17 | 6 (35.3) | 0.81 (0.27-2.21) | - |
| Treatment history | New | 292 | 122 (41.6) | Reference | - |
|  | Relapse | 15 | 3 (20.0) | 0.35 (0.08-1.13) | - |
|  | Retreatment | 5 | 2 (50.0) | 1.40 (0.17-11.81) | - |
|  | Other | 0 | 0 (NaN) | - | - |
| Diagnostic delay | No | 158 | 61 (38.4) | Reference | - |
|  | Yes | 154 | 66 (43.1) | 1.22 (0.78-1.92) | - |
| HIV status | HIV negative | 268 | 101 (37.7) | Reference | - |
|  | HIV positive | 43 | 25 (58.1) | 2.30 (1.20-4.47) | - |
|  | HIV test not done | 1 | 1 (100.0) | 3502337.19 (0.00-NA) | - |
|  | Status unknown | 0 | 0 (NaN) | - | - |
| Hospitalization | Not hospitalized | 203 | 60 (29.6) | Reference | Reference |
|  | Hospitalized | 109 | 67 (61.5) | 3.80 (2.34-6.24) | 3.39 (1.98-5.87) |
| Weight loss | No | 31 | 3 (9.7) | Reference | Reference |
|  | Yes | 281 | 124 (44.1) | 7.37 (2.54-31.32) | 7.79 (2.33-36.41) |
| Current appetite | Good appetite | 89 | 25 (28.1) | Reference | - |
|  | Poor appetite | 223 | 102 (45.7) | 2.16 (1.28-3.72) | - |
| Change in appetite | Increase food intake | 11 | 6 (54.5) | Reference | - |
|  | No decrease food intake | 79 | 23 (29.1) | 0.34 (0.09-1.24) | - |
|  | Moderate decrease food intake | 152 | 56 (37.1) | 0.49 (0.14-1.70) | - |
|  | Severe decrease food intake | 70 | 42 (59.2) | 1.21 (0.32-4.38) | - |
| Below international poverty line before having TB | No | 287 | 114 (39.6) | Reference | - |
|  | Yes | 25 | 13 (54.2) | 1.80 (0.78-4.24) | - |
| Below international poverty line at the time of TB diagnosis | No | 262 | 98 (37.3) | Reference | Reference |
|  | Yes | 50 | 29 (59.2) | 2.44 (1.32-4.60) | 2.58 (1.28-5.33) |
