# Supplementary material for: The prevalence of undernutrition and associated risk factors in people with tuberculosis in Lao People’s Democratic Republic
Source: PLoS One. 2025 Jun 20;20(6):e0324838. doi: 10.1371/journal.pone.0324838 (PMC12180643; doi:10.1371/journal.pone.0324838)
Supplement: Attachment — Submitted filename: R1_responses to reviewers_v1.docx [file pone.0324838.s003.docx]

**Thank you for your further review and comments. We added our responses to each comment with red font text and revised the manuscript with track changes accordingly.**

**Reviewer #1:** The reported odds ratios and their corresponding log(odds ratios) are inconsistent and require re-analysis. Ensure that computations align correctly before finalizing the results.
(Figure 1):
o The odds ratio (OR) quantifies the association between an exposure and an outcome. The null hypothesis (no association) corresponds to an OR of 1, meaning the odds of the outcome are the same in both groups.
o Since the logarithm of 1 is 0 (i.e., log(1) = 0), the null value for the log odds ratio (log(OR)) is also 0.
o Your figure shows that all variables have Log(OR) > 0, suggesting that all are positively associated with the outcome. However, this contradicts the textual interpretation of the results using odds ratios.

Thank you for your clarifications. The results are estimated with antilog scale and therefore, for all the results shown in fig 1, the null hypothesis of the analysis still corresponds to an OR of 1, not zero. Given that a variable (weight decreased) has much a wider confidence interval (i.e. CI 2.3-36.4 for hospitalization), we applied a log scale to the x-axis label (not results themselves) only for the visualization purposes. Hence, we do not change the analytical approach.

We understand that there might be confusions as raised by the reviewer, and therefore we revised the axis title and also added a footnote for this matter.

o Additionally, the antilog of the reported log(OR) values does not match the stated odds ratios. For example, the antilog of 3.1 does not yield an OR of 6.8 for the age group 15–24. A re-analysis is necessary.

Thank you for spotting these inconsistencies. We revised the paragraph in result section accordingly.

… in **Figure 1** (results with univariate and multivariate analysis are available in **Table S1** in appendix). There were substantially heightened risk of low BMI among different age groups. Specifically, individuals aged 15-24 years old exhibited a remarkably higher risk (AOR: 6.9, 95%CI: 2.2-23.2) compared to those aged 35-44 years old. ~~Similarly, the risk remained elevated for those aged 25-34 years old (AOR: 2.2, 95%CI: 0.9-5.5) and even for individuals aged 65 years and above (AOR=2.0, 95% CI: 0.8-4.8)~~. The presence of drug resistance also emerged as a significant risk factor for low BMI (AOR: 3.2, 95% CI=1.0-11.8). Moreover, patients who were hospitalized until the diagnosis of TB faced a substantially higher risk of low BMI (AOR: 3.4, 95% CI=2.0-5.9). This indicates that the severity of the TB condition, requiring hospitalization, is associated with a greater likelihood of low BMI at the time of TB diagnosis. Notably, individuals who experienced weight loss in the last 6 months prior to TB diagnosis were at a significantly elevated risk of having a low BMI (AOR: 7.8, 95% CI: 2.3-36.4). Finally, patients who were categorized as being below the international poverty line at the time of TB diagnosis also faced an increased risk of low BMI (AOR: 1.9, 95% CI 1.0-3.6).

*x-axis is with log-scale.

**Reviewer #2:** This study investigates malnutrition among tuberculosis patients at the time of diagnosis in Lao PDR. The results and discussion are presented based on data obtained from central and provincial hospitals in relation to the stated research objectives. Although the representativeness of the collected data in tuberculosis patients is a concern, the study appropriately discusses its limitations. Given its valuable insights into the topic, this manuscript is considered worthy of acceptance.

Thanks for your favourable comment and decision to our manuscript.
